# GABAPENTIN TREATMENT FOR CHALLENGING BEHAVIORS IN AUTISM SPECTRUM DISORDER AND INTELLECTUAL DISABILITY: A CASE REPORT

**DOI:** 10.1192/j.eurpsy.2023.1891

**Published:** 2023-07-19

**Authors:** S. Marini, L. D’Agostino, A. Gentile

**Affiliations:** Mental Health, National Heath Service, Termoli, Italy

## Abstract

**Introduction:**

Autism Spectrum Disorder (ASD) includes a group of developmental disabilities characterized by patterns of delay and deviance in the development of social, communicative, cognitive skills and the presence of repetitive and stereotyped behaviors as well as restricted interests (APA, 2013 DSM 5th ed.).

**Objectives:**

A 22-years old male outpatient affected by Autism Spectrum Disorder (Level 3) and severe ID presented serious challenging behaviors. The patient did not suffer from other psychiatric or neurologic pathologies. The patient did not have constipation or diarrhea or painful symptoms. The patients assumed carbamazepine (modified release) 800 mg/day (blood dosage 6,8 microgram/ml), clonazepam 2,5 mg/ml 15 drops/day, lorazepam 7,5 mg/die.

**Methods:**

Due to the onset of challenging behaviors risperidone was introduced. At the dosage of 2 mg/day, the patient reached a discrete control of challenging behaviors. After stopping risperidone because of oculogyric crisis, the patient started to assume valproic acid (chronic formulation) up to 1000 mg/day. After three weeks the patient presented an increase in the blood dosage of ammonium. After the drug stop, the patient began to re-present challenging behaviors. The authors decided to add topiramate at a dosage of 25 mg per day. After three days, the patient began to present nocturnal urinary incontinence. Topiramate was stopped and Gabapentin was introduced in the treatment up to the dosage of 900 mg/day. Lorazepam was gradually tapered off until the intake was terminated, and clonazepam was reduced to 5 drops/day taken at bedtime. The dosage of carbamazepine remained stable.

**Results:**

**Table 1.** Behavior Problems Inventory subscales scores

	**Pre-treatment (T0)**	**Post-treatment (T1)**	**% Improvements**			
Behavior Problems Inventory Subscales	Frequency	Severity	Frequency	Severity	Frequency	Severity
Self-Injurious Behavior	6	2	5	2	16,7%	0%
Stereotyped Behavior	49	19	38	16	22,5%	15,8%
Aggressive/destructive Behavior	39	25	24	20	38,5%	20%

**Conclusions:**

According to the GABAergic hypothesis of ASD, inhibitory signaling of GABA within and between cortical minicolumns appears to be altered. This alteration would result in information processing with high discrimination between correlated stimuli rather than a generalization of them (Casanova et al. Neuroscientist 2003; 9: 496-507). Gabapentin is a ligand of the auxiliary alpha-2-delta subunit site of voltage-dependent calcium channels and acts as an inhibitor of the channel (Sills Curr Opin Pharmacol 2006; 6 (1):108-13). The altered expression of alpha-2-delta 1 or alpha-2-delta 3 can cause a chronic imbalance between arousal and inhibition that is quite characteristic of ASD (Nelson et al. Neuron 2015;87:684-698). The authors want to speculate on a hypothetical function of gabapentin in remodeling the expression of alpha-2-delta subunits in people with autism and the processing of neural information.

**Disclosure of Interest:**

None Declared

